# Impact of the COVID-19 pandemic on critical care utilization in Japan: a nationwide inpatient database study

**DOI:** 10.1186/s40560-022-00645-0

**Published:** 2022-12-02

**Authors:** Hiroyuki Ohbe, Yusuke Sasabuchi, Hiroki Matsui, Hideo Yasunaga

**Affiliations:** 1 Department of Clinical Epidemiology and Health Economics, School of Public Health, The University of Tokyo, 7-3-1 Hongo, Bunkyo-ku, Tokyo, 113-0033 Japan; 2 Data Science Center, Jichi Medical University, 3311-1 Yakushiji, Shimotsuke-shi, Tochigi-ken 329-0498 Japan

**Keywords:** COVID-19 pandemic, Critical care utilization, Bed occupancy, Interrupted time-series analysis, High-dependency care unit

## Abstract

**Background:**

The coronavirus disease 2019 (COVID-19) pandemic has disrupted critical care services worldwide. Examining how critical care systems responded to the COVID-19 pandemic on a national level will be useful in setting future critical care plans. The present study aimed to describe the utilization of critical care services before and during the COVID-19 pandemic using a nationwide Japanese inpatient administrative database.

**Methods:**

All patients admitted to an intensive care unit (ICU) or a high-dependency care unit (HDU) from February 9, 2019, to February 8, 2021, in the Japanese Diagnosis Procedure Combination inpatient database were included. February 9, 2020, was used as the breakpoint separating the periods before and during COVID-19 pandemic. Hospital and patient characteristics were compared before and during the COVID-19 pandemic. Change in ICU and HDU bed occupancy before and during the COVID-19 pandemic was evaluated using interrupted time-series analysis.

**Results:**

The number of ICU patients before and during the COVID-19 pandemic was 297,679 and 277,799, respectively, and the number of HDU patients was 408,005 and 384,647, respectively. In the participating hospitals (383 ICU-equipped hospitals and 460 HDU-equipped hospitals), the number of hospitals which increased the ICU and HDU beds capacity were 14 (3.7%) and 33 (7.2%), respectively. Patient characteristics and outcomes in ICU and HDU were similar before and during the COVID-19 pandemic except main etiology for admission of COVID-19. The mean ICU bed occupancy before and during the COVID-19 pandemic was 51.5% and 47.5%, respectively. The interrupted time-series analysis showed a downward level change in ICU bed occupancy during the COVID-19 pandemic (− 4.29%, 95% confidence intervals − 5.69 to − 2.88%), and HDU bed occupancy showed similar trends. Of 383 hospitals with ICUs, 232 (60.6%) treated COVID-19 patients in their ICUs. Their annual hospital case volume of COVID-19 ICU patients varied greatly, with a median of 10 (interquartile range 3–25, min 1, max 444).

**Conclusions:**

The ICU and HDU bed capacity did not increase while their bed occupancy decreased during the COVID-19 pandemic in Japan. There was no change in clinicians’ decision-making to forego ICU/HDU care for selected patients, and there was no progress in the centralization of critically ill COVID-19 patients.

**Supplementary Information:**

The online version contains supplementary material available at 10.1186/s40560-022-00645-0.

## Introduction

The coronavirus disease 2019 (COVID-19) pandemic has contributed to substantial disruptions in the delivery of healthcare services around the world [1, 2]. The rapid rise in patients with COVID-19 requiring hospitalization quickly overwhelmed acute care services of health systems and imposed a high staffing burden at least four times more than usual, including critical care [3–5].

In Japan, the first patient with COVID-19 was reported on January 16, 2020 [6], and the first patient with COVID-19 admitted to the intensive care unit (ICU) was confirmed on February 9, 2020 [7]. Since then, the number of COVID-19 cases in Japan has continued to rise with three pandemic waves where, by the end of February 2021, there had been 432,773 laboratory-confirmed COVID-19 cases and 7887 COVID-19-related deaths in Japan [8]. To prevent the collapse of the healthcare delivery system, the Japanese government invested a large amount of money in the healthcare system and announced states of COVID-19 emergency from April 7, 2020, to May 25, 2020, and from January 8, 2021, to March 21, 2021 [8].

To date, there are no studies describing critical care utilization in Japan during COVID-19 at a national level. The reported number of ICU beds per 100,000 population in Japan is about five, which is less than that in most other developed countries [9–11]. Given the increasing strain forced by COVID-19 and the relatively low availability of ICU beds in Japan, examining how the critical care system at a national level responded to the disastrous conditions of the COVID-19 pandemic will be useful in setting future critical care plans. Therefore, the present study aimed to compared the utilization of critical care services during the COVID-19 pandemic to that before the pandemic using a nationwide inpatient database.

## Methods

### Data source

This was a retrospective cohort study that used routinely collected nationwide inpatient administrative data in Japan to compare the utilization of critical care services during the COVID-19 pandemic to that before the pandemic. The Institutional Review Board of The University of Tokyo approved this study (approval number, 3501-3). No information allowing the identification of individual patients, hospitals, or physicians was obtained, and the requirement for informed consent was waived because of the anonymous nature of the data.

We used the Japanese Diagnosis Procedure Combination inpatient database (DPC database), which contains discharge abstracts and administrative claims data from more than 1500 participating public and private acute-care hospitals in Japan that voluntarily contribute to the database [12]. The database includes the following patient-level data for all hospitalizations: age, sex, route of admission, cognitive function before admission, admission type, diagnoses recorded using *International Classification of Diseases, Tenth Revision* (ICD-10) codes, daily procedures recorded using Japanese medical procedure codes, daily drug administrations, and discharge status. In a previous study examining the validity of the recorded procedures and diagnoses, the sensitivity and specificity of procedures exceeded 90%, whereas the sensitivity and specificity of the primary diagnoses were 78.9% and 93.2%, respectively [13].

We also used the Survey of Medical Institutions 2019 and 2020, which include the facilities’ information and statistics as of July 1, 2019 and July 1, 2020, respectively [14]. We combined this information with the data in the Japanese Diagnosis Procedure Combination inpatient database using a specific hospital identifier. The Survey of Medical Institutions included academic hospitals, tertiary emergency hospitals, types of wards [e.g., general, ICU, or high-dependency care units (HDUs)], and the number of licensed hospital beds in each ward.

### Before and during the COVID-19 pandemic

In Japan, the first COVID-19-related ICU admission case was confirmed on February 9, 2020 [7]. Therefore, in this study, February 9, 2020, was used as the breakpoint separating the periods before and during COVID-19 into two 1-year periods [15]: before the COVID-19 pandemic from February 9, 2019, to February 8, 2020, and during the COVID-19 pandemic from February 9, 2020, through February 8, 2021.

### Study population

We examined two separate cohorts of patients in ICUs, as well as HDUs, which are potential alternatives to ICUs [16, 17]. For the ICU patients, we included all patients admitted to an ICU between February 9, 2019, and February 8, 2021, from hospitals participating in the DPC database that also had records in the Survey of Medical Institutions and had at least one ICU bed reported in the Survey of Medical Institutions of both 2019 and 2020. In this study, an ICU was defined as a separate unit providing critical care services with at least one physician on site 24 h per day, around-the-clock nursing, the equipment necessary to care for critically ill patients, and a nurse-to-patient ratio of > 1:2 [18]. As for HDU patients, we included all patients admitted to an HDU between February 9, 2019, and February 8, 2021, from hospitals participating in the DPC database that also had records in the Survey of Medical Institutions and had at least one HDU bed in the Survey of Medical Institutions of both 2019 and 2020. The definition of an HDU in this study was almost the same as an ICU, except that the required nurse-to-patient ratio was 1:3, 1:4, or 1:5. The Japanese medical procedure codes to define ICU and HDU are listed in Additional file 1: Table S1. We did not include patients admitted to the neonatal ICU or obstetric ICU in this study.

### Statistical analysis

This study presents hospital characteristics before and during the COVID-19 pandemic. Hospital characteristics were based on the Survey of Medical Institutions 2019 before the COVID-19 pandemic and the Survey of Medical Institutions 2020 during the COVID-19 pandemic. We then compared the patient characteristics and outcomes before and during the COVID-19 pandemic. Due to the large sample size in this study, the patient characteristics and outcomes were compared using standardized mean differences, with an absolute standardized mean difference of ≤ 10% denoting a negligible imbalance between the two groups [19]. Patient characteristics included age, sex, route of admission, cognitive function before admission, admission type, main etiologies for admission, and organ support at least once during ICU/HDU stay. Admission type was categorized as elective surgery, emergency surgery, or nonsurgical/acute medical problem. We defined patients who were admitted to the ICU/HDU on the day of elective or emergency surgery with general anesthesia as elective or emergency surgery patients. Main etiologies for admission were defined by the ICD-10 codes in the admission-precipitating diagnosis as follows: circulatory diseases (ICD-10 codes: I00-I99), neoplasms and diseases of the blood (C00-D89), injury, poisoning and external causes (S00-T98), abdominal diseases (K00-K93), respiratory diseases (J00-J99), COVID-19 (U071), and others. We also performed a descriptive epidemiology of COVID-19 patients. Continuous variables are presented as means and standard deviation (SD) or as medians and interquartile ranges (IQRs), as appropriate. Categorical variables are presented as frequencies and percentages. All analyses were performed using Stata/MP, Version 16.0 (StataCorp, College Station, TX, USA).

### Analysis of occupancy

We calculated the daily occupancy of ICUs/HDUs by all patients in total, patients with invasive mechanical ventilation (IMV), and patients with extracorporeal membrane oxygenation (ECMO). We defined occupancy as the total number of relevant reimbursements in the cohort on a given day divided by the total number of licensed ICU/HDU beds in the participating hospitals. Change in occupancy before and during the COVID-19 pandemic was evaluated using patient-level segmented linear regression with interrupted time-series analysis [20]. The equation for the interrupted time-series analysis was as follows:

$${Y}_{t}={\beta }_{0}+{\beta }_{1}T+{\beta }_{2}{X}_{t}+{\beta }_{3}T{X}_{t},$$

where ${Y}_{t}$ is the occupancy, $T$ is the month since the beginning of the study period (coded from 1 to 24), and ${X}_{t}$ is a dummy variable indicating before or during the COVID-19 pandemic (coded 0 or 1). In this model, ${\beta }_{2}$ represents the level change immediately during the COVID-19 pandemic and ${\beta }_{3}$ represents the trend change during the COVID-19 pandemic compared to the baseline trend before the COVID-19 pandemic. As the sensitivity analyses for controlling for seasonality, we performed the interrupted time-series analyses stratified by the calendar month [20].

## Results

There were 612 ICU-equipped hospitals with 7097 ICU beds in Japan as reported by the Survey of Medical Institutions 2019, while there were 597 ICU-equipped hospitals with 7026 ICU beds reported by the Survey of Medical Institutions 2020. After accounting for our inclusion criteria, the study cohort consisted of 573,102 patients admitted to 383 hospitals with 4945 ICU beds before the COVID-19 pandemic and 4984 ICU beds during the COVID-19 pandemic (Additional file 1: Fig. S1). Thus, 70% (4945/7097 beds) of all ICU beds in 2019 and 71% (4984/7026 beds) of all ICU beds in 2020 were included in the present study. The median number of ICU beds was 10 (IQR 8–16), and 14 (3.7%) hospitals increased their ICU beds during the COVID-19 pandemic (Table 1). The mean annual hospital revenue from ICU patients was 2400 (SD 1900) before the COVID-19 pandemic, and it decreased to 2200 (SD 1700) million yen after the pandemic, which corresponds to a difference of approximately 200 million yen.

**Table 1:** Characteristics of hospitals equipped with ICU and HDU beds

Hospital characteristics	Hospitals with ICU beds		Hospitals with HDU beds	
	Before COVID-19 pandemic (*n* = 383)	During COVID-19 pandemic (*n* = 383)	Before COVID-19 pandemic (*n* = 460)	During COVID-19 pandemic (*n* = 460)
Academic hospital, *n* (%)	76 (19.8%)	76 (19.8%)	62 (13.5%)	63 (13.7%)
Tertiary emergency hospital, *n* (%)	190 (49.6%)	191 (49.9%)	201 (43.7%)	203 (44.1%)
Number of acute hospital beds, median (IQR)	468 (352–588)	465 (340–588)	396 (265–552)	391 (265–547)
Hospital with ICU beds, *n* (%)	383 (100%)	383 (100%)	276 (60.0%)	275 (59.8%)
Number of ICU beds, beds				
Total	4945	4983	3877	3909
Median (IQR)	10 (8–16)	10 (8–16)	10 (8–18)	10 (8–18)
Hospitals with increased ICU beds from 2019 to 2020, *n* (%)	–	14 (3.7%)	–	9 (2.0%)
Hospital with HDU beds, *n* (%)	276 (72.1%)	279 (72.8%)	460 (100%)	460 (100%)
Number of HDU beds, beds				
Total	5863	6174	7969	8311
Median (IQR)	20 (10–28)	20 (10–28)	14 (8–24)	14 (8–24)
Hospitals with increased HDU beds from 2019 to 2020, *n* (%)	–	21 (5.5%)	–	33 (7.2%)
Annual hospital case volume of ICU/HDU patients*				
Total	297,679	277,799	408,005	384,647
Mean (SD)	811 (597)	753 (570)	911 (747)	866 (696)
Annual hospital revenue from ICU/HDU patients, million yen*				
Total	877,000	804,000	707,000	660,000
Mean (SD)	2,400 (1,900)	2,200 (1,700)	1,600 (1,400)	1,500 (1,300)

*ICU* intensive care unit; *HDU* high-dependency care unit; *COVID-19* coronavirus disease 2019; *IQR* interquartile range; *HDU* high-dependency care unit; *SD* standard deviation

*Annual hospital case volume and annual hospital revenue were calculated by using only ICU patients for hospitals with ICU beds and only HDU patients for hospitals with HDU beds

Of 573,102 eligible patients, 297,679 and 277,799 patients were admitted to the ICU before and during the COVID-19 pandemic, respectively (Table 2). The mean age of patients was 67.3 (SD = 18.7) and 67.8 (SD = 18.3) before and during the COVID-19 pandemic, respectively. About 60% were male in both periods. About one-third of patients were admitted to the ICU after elective surgery in both periods. In-hospital mortality rates were 12.0% and 12.1% before and during the COVID-19 pandemic, respectively. Patient characteristics and outcomes were also comparable before and during the COVID-19 pandemic except for the main etiology of ICU admission being due to COVID-19.

**Table 2:** Characteristics and outcomes of ICU and HDU patients

Characteristics and outcomes	ICU patients			HDU patients		
	Before COVID-19 pandemic (*n* = 297,679)	During COVID-19 pandemic (*n* = 275,423)	SMD, %	Before COVID-19 pandemic (*n* = 408,005)	During COVID-19 pandemic (*n* = 384,647)	SMD, %
Age, year, mean (SD)	67.3 (18.7)	67.8 (18.3)	2.7	70.9 (17.4)	71.2 (17.3)	0.1
Male, %	180,260 (60.6%)	167,806 (60.9%)	0.8	232,993 (57.1%)	220,091 (57.2%)	0.2
Route of admission, %						
Home	268,903 (90.3%)	249,076 (90.4%)	− 0.8	359,513 (88.1%)	338,379 (88.0%)	− 0.9
Another hospital	21,661 (7.3%)	19,562 (7.1%)		23,191 (5.7%)	21,595 (5.6%)	
Nursing home	7113 (2.4%)	6785 (2.5%)		25,298 (6.2%)	24,672 (6.4%)	
Dementia, %						
None	263,892 (88.6%)	241,643 (87.7%)	− 2.8	321,092 (78.7%)	299,986 (78.0%)	− 1.9
Mild dementia	21,533 (7.2%)	21,404 (7.8%)		46,577 (11.4%)	44,593 (11.6%)	
Moderate-to-severe dementia	12,254 (4.1%)	12,376 (4.5%)		40,336 (9.9%)	40,068 (10.4%)	
Admission type, *n* (%)						
Elective surgery	102,700 (34.5%)	95,001 (34.5%)	− 0.1	67,826 (16.6%)	59,879 (15.6%)	− 3.0
Emergency surgery	33,125 (11.1%)	30,545 (11.1%)		29,472 (7.2%)	27,459 (7.1%)	
Nonsurgical/acute medical problem	161,852 (54.4%)	149,877 (54.4%)		310,704 (76.2%)	297,308 (77.3%)	
Main etiologies for admission, *n* (%)						
Circulatory diseases	132,736 (44.6%)	120,295 (43.7%)	− 1.8	163,073 (40.0%)	150,981 (39.3%)	− 1.5
Neoplasms and diseases of the blood	73,572 (24.7%)	68,601 (24.9%)	0.4	62,604 (15.3%)	56,448 (14.7%)	− 1.9
Injury, poisoning and external causes	19,570 (6.6%)	17,747 (6.4%)	− 0.5	47,490 (11.6%)	42,618 (11.1%)	− 1.8
Abdominal diseases	17,659 (5.9%)	16,388 (6.0%)	0.1	36,911 (9.0%)	33,466 (8.7%)	− 1.2
Respiratory diseases	12,721 (4.3%)	10,168 (3.7%)	− 3.0	33,973 (8.3%)	26,450 (6.9%)	− 5.5
COVID-19	0 (0.0%)	5131 (1.9%)	19.5	0 (0.0%)	18,888 (4.9%)	32.1
The others	41,419 (13.9%)	37,093 (13.5%)	− 1.3	63,951 (15.7%)	55,795 (14.5%)	− 3.3
Organ support during ICU/HDU stay, *n* (%)						
Invasive mechanical ventilation	98,700 (33.2%)	87,603 (31.8%)	− 2.8	53,026 (13.0%)	48,752 (12.7%)	− 1.0
Noradrenaline	92,024 (30.9%)	88,533 (32.1%)	2.6	33,490 (8.2%)	34,792 (9.0%)	3.0
Continuous renal replacement therapy	14,592 (4.9%)	12,824 (4.7%)	− 1.2	3492 (0.9%)	3283 (0.9%)	0.0
Intra-aortic balloon pumping	8360 (2.8%)	7532 (2.7%)	− 0.4	2007 (0.5%)	1875 (0.5%)	− 0.1
Extracorporeal membrane oxygenation	4064 (1.4%)	3701 (1.3%)	− 0.2	750 (0.2%)	669 (0.2%)	− 0.2
Left ventricular assist device	22 (0.0%)	30 (0.0%)	0.4	2 (0.0%)	1 (0.0%)	− 0.1
Intracranial pressure monitoring	977 (0.3%)	813 (0.3%)	− 0.6	366 (0.1%)	316 (0.1%)	− 0.3
ICU/HDU mortality, *n* (%)	17,770 (6.0%)	17,023 (6.2%)	0.9	23,557 (5.8%)	23,851 (6.2%)	1.8
Discharge status, *n* (%)						
In-hospital mortality	35,714 (12.0%)	33,386 (12.1%)	− 0.8	48,306 (11.8%)	46,448 (12.1%)	− 0.8
Home	201,418 (67.7%)	186,582 (67.7%)		248,489 (60.9%)	233,694 (60.8%)	
Another hospital	56,433 (19.0%)	51,521 (18.7%)		93,782 (23.0%)	87,916 (22.9%)	
Nursing home	4112 (1.4%)	3934 (1.4%)		17,425 (4.3%)	16,588 (4.3%)	
Length of hospital stay, days, median (IQR)	17 (10–31)	16 (9–30)	− 5.1	14 (7–27)	14 (7–25)	− 4.4
Length of ICU/HDU stay, days, median (IQR)	2 (1–4)	2 (1–4)	− 1.3	2 (1–4)	2 (1–4)	3.3
Hospitalization costs, million yen, mean (SD)	2.1 (1.3–3.7)	2.1 (1.4–3.7)	− 0.9	1.3 (0.7–2.1)	1.3 (0.7–2.1)	− 1.0

*ICU* intensive care unit; *HDU* high-dependency care unit; *COVID-19* coronavirus disease 2019; *SMD* standardized mean difference; *SD* standard deviation; *IQR* interquartile range

**Fig. 1:**
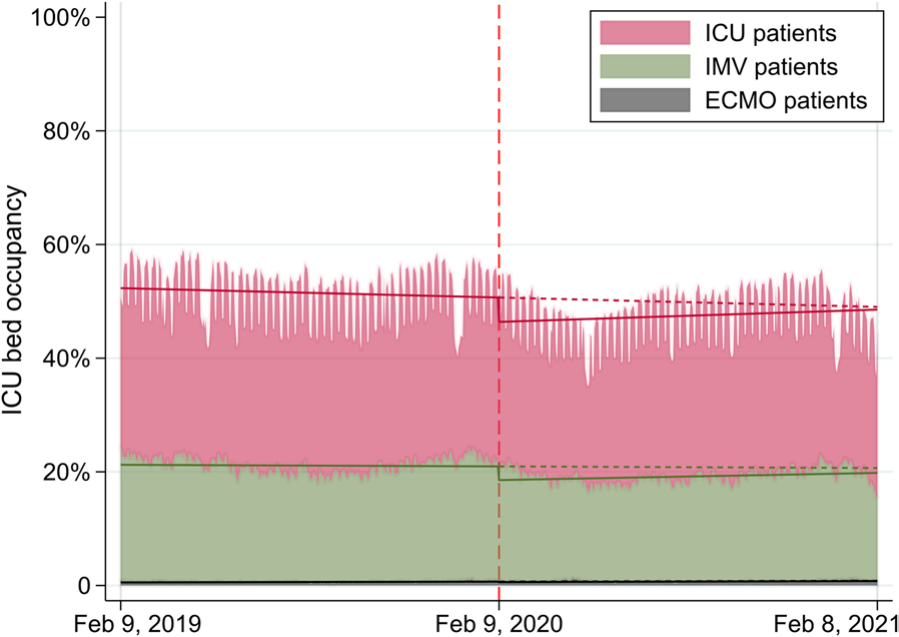
Change in ICU bed occupancy before and during the COVID-19 pandemic. *ICU* intensive care unit; *COVID-19* coronavirus disease 2019; *IMV* invasive mechanical ventilation; *ECMO* extracorporeal membrane oxygenation. Solid and dotted lines represent the predicted outcomes using interrupted time-series analysis. Red dashed line indicates the start of the COVID-19 pandemic on February 9, 2020

The mean ICU bed occupancy before the COVID-19 pandemic (51.5%) was greater than that during the COVID-19 pandemic (47.5%) (Table 3, Fig. 1). The interrupted time-series analysis showed a downward level of change in ICU bed occupancy immediately during the COVID-19 pandemic (− 4.29%, 95% confidence intervals [CIs] − 5.69 to − 2.88%) followed by a small upward slope change (0.31% change in trend per month; 95% CI 0.11–0.51%). The results of interrupted time-series analysis showed that the ICU bed occupancy by patients with invasive mechanical ventilation, patients with ECMO, and patients without any organ support were similar.

**Table 3:** The results of interrupted time-series analysis for ICU and HDU patients

Variables	Before COVID-19 pandemic	During COVID-19 pandemic	Level change, % (95% CIs)	Trend change, % per month (95% CIs)
ICU				
ICU bed occupancy, %	51.5	47.5	− 4.29 (− 5.69, − 2.88)	0.31 (0.11, 0.51)
Invasive mechanical ventilation, %	21.1	19.2	− 2.40 (− 2.80, − 2.01)	0.13 (0.07, 0.18)
Extracorporeal membrane oxygenation, %	0.6	0.7	− 0.08 (− 0.13, − 0.04)	0.00 (0.00, 0.01)
Without any organ support, %	24.1	22.3	− 1.58 (− 2.41, − 0.75)	0.17 (0.05, 0.29)
HDU				
HDU bed occupancy, %	46.9	44.8	− 8.12 (− 9.02, − 7.22)	1.23 (1.10, 1.36)
Invasive mechanical ventilation, %	6.5	5.6	− 1.44 (− 1.64, − 1.24)	0.05 (0.02, 0.08)
Extracorporeal membrane oxygenation, %	0.0	0.1	0.00 (− 0.01, 0.00)	0.00 (0.00, 0.00)
Without any organ support, %	38.8	37.6	− 6.46 (− 7.22, − 5.70)	1.16 (1.05, 1.26)

*ICU* intensive care unit; *HDU* high-dependency care unit; *COVID-19* coronavirus disease 2019; *CI* confidence interval

During the COVID-19 pandemic, a total of 5131 COVID-19 patients were admitted to ICUs. In-hospital mortality was 16.2% (831/5131), and the median length of ICU stay was 6 days (IQR 2–12 days) (Additional file 1: Table S2). There were three pandemic waves of COVID-19 ICU patients during the observation period (Additional file 1: Fig. S2). The maximum daily number of COVID-19 patients treated in the ICUs was 351 on January 25, 2021, which represented 7.0% (351/4984) of ICU beds in 2020. Of 383 hospitals equipped with ICUs, about 150 hospitals treated COVID patients in their ICUs immediately during the first wave of the COVID-19 pandemic, and 232 (60.6%) hospitals treated COVID-19 patients in their ICUs within 1 year of the beginning of the COVID-19 pandemic (Additional file 1: Fig. S3). Meanwhile, 151 (39.4%) hospitals did not treat any COVID-19 patients in their ICUs during the 1st year of the COVID-19 pandemic. In the 232 hospitals that were equipped with ICUs and treated COVID-19 patients in their ICUs, the annual hospital case volume of COVID-19 ICU patients varied greatly, with a median of 10 (IQR 3–25, min 1, max 444) (Additional file 1: Fig. S4).

**Fig. 2:**
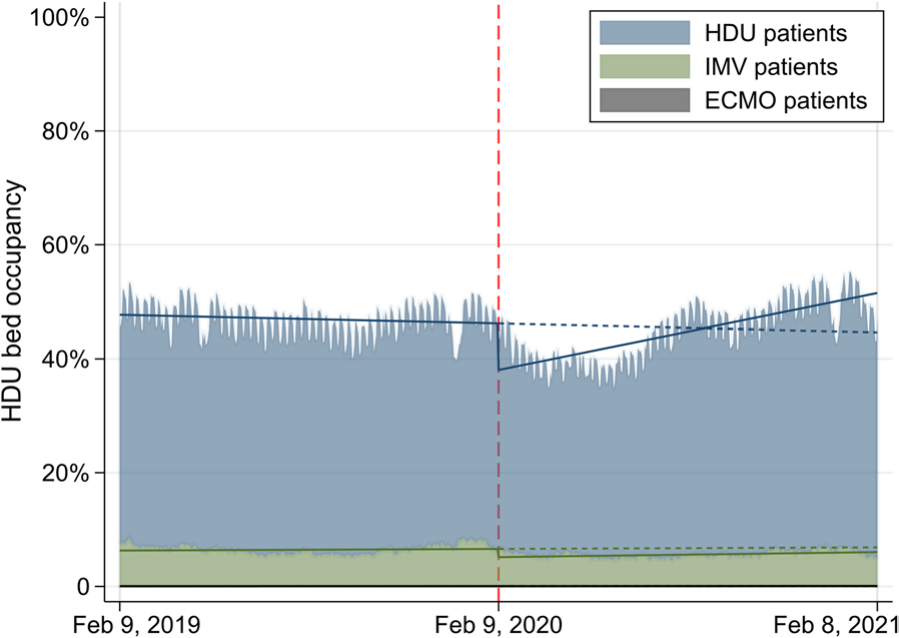
Change in HDU bed occupancy before and during the COVID-19 pandemic. *HDU* high-dependency care unit; *COVID-19* coronavirus disease 2019; *IMV* invasive mechanical ventilation; *ECMO* extracorporeal membrane oxygenation. Solid and dotted lines represent the predicted outcomes using interrupted time-series analysis. Red dashed line indicates the start of the COVID-19 pandemic on February 9, 2020

The study cohort of HDU patients consisted of 792,652 patients admitted to 460 hospitals with 7969 HDU beds before the COVID-19 pandemic and 8311 HDU beds during the COVID-19 pandemic, representing 62% (7969/12,949 beds) of all HDU beds in 2019 and 61% (8311/13,546 beds) of all HDU beds in 2020 (Table 1, Additional file 1: Fig. S5). The median number of HDU beds was 14 (IQR 8–24) in 2020, and 33 (7%) hospitals increased their HDU beds from 2019 to 2020. Characteristics and outcomes of HDU patients were similar before and during the COVID-19 pandemic except for the main etiology for ICU admission being due to COVID-19 (Table 2). The mean HDU bed occupancy before the COVID-19 pandemic (46.9%) was greater than that during the pandemic (44.8%) (Table 3, Fig. 2). The interrupted time-series analysis of HDU bed occupancy showed a downward level of change immediately after the COVID-19 pandemic (-8.12%, 95% confidence intervals [CIs] − 9.02 to − 7.22%) followed by an upward slope change (1.23% change in trend per month; 95% CI 1.10–1.36%). The results of sensitivity analyses for controlling for seasonality were similar with those in the main analyses (Additional file 1: Table S3).

During the COVID-19 pandemic, a total of 18,888 COVID-19 patients were admitted to an HDU, and their in-hospital mortality was 7.6% (1431/18,888) (Additional file 1: Table S2). The maximum daily number of COVID-19 patients treated in HDUs was 1179 on January 18, 2021, which represented 14.2% (1179/8311) of all HDU beds in 2020 (Additional file 1: Fig. S6). Of 460 hospitals equipped with HDUs, 292 (63.5%) hospitals treated COVID-19 patients in their HDUs within 1 year of the COVID-19 pandemic start, while 168 (36.5%) hospitals did not treat any COVID-19 patients during that period (Additional file 1: Fig. S7). In the 292 hospitals that were equipped with HDUs and treated COVID-19 patients in their HDUs, the annual hospital case volume of COVID-19 HDU patients varied greatly, with a median of 20 (IQR 5–82, min 1, max 1434) (Additional file 1: Fig. S8).

## Discussion

The present study describes in detail the utilization of critical care services in Japan at a national level before and during the COVID-19 pandemic and presents several key findings. First, most hospitals with ICUs and HDUs did not increase the bed capacity of their licensed critical care beds in response to COVID-19 by July 1, 2020. Second, the number of ICU/HDU admissions and that of ICU/HDU bed occupancies declined significantly during the COVID-19 pandemic. Due to the decrease in ICU patients, hospitals experienced a decrease in revenue of 200 million yen per year on average. Third, there was no change in clinicians’ decision-making to forego ICU care for selected patients in Japanese ICUs. The ICU/HDU bed occupancy by patients requiring organ support such as IMV and ECMO decreased, which was also the case for patients undergoing elective surgeries. Mortality and length of ICU stay remained unchanged before and during COVID-19. Fourth, within 1 year from the start of the COVID-19 pandemic, the number of COVID patients treated in the ICUs was still low, and about 40% of ICUs did not treat any COVID-19 patients. The hospital volume of COVID-19 patients in the ICU was very limited in most hospitals, and patients were dispersed among many ICUs. A significant number of COVID-19 patients were treated in HDUs, including those who required organ support.

### In the context of prior literature

The previous study in Alberta, Canada, where the critical care surge of COVID-19 did not exceed the ICU bed capacity, showed the substantial decline of ICU admissions during COVID-19 lockdown [21]. However, contrary to the results of this study, they reported marked reductions in the numbers of ICU admissions for elective surgery and avoidable waiting time before ICU discharge during the COVID-19 pandemic compared with those during historical periods. Notably, this adequate critical care utilization was achieved despite no concomitant policy change in ICU admission criteria, and the authors hypothesized that lockdown had a major contribution to this effect. These same trends of reduced ICU admissions were also reported in pediatric ICUs [22, 23]. Nevertheless, further data on the impact of critical care utilizations during the COVID-19 pandemic are limited, and further studies are needed to elucidate this effect.

### Implications for clinicians and policy

There are several possible options for responding to surges in demand for care for critically ill patients during a pandemic, including critical care triage, rationing of resources, provision of intensive care outside the ICU, rapidly building new hospitals with ICUs, and centralization of critically ill patients in designated hospitals with ICUs [24, 25]. Guidelines for critical care utilization during the COVID-19 pandemic recommend reorganizing critical care structure in readiness for potential surges in COVID-19-related critical illness and suspending elective medical and surgical procedures once ongoing chains or community transmission of COVID-19 has been documented within a State/Province/Country [26, 27]. Combined with the results of this study describing critical care utilization before and during COVID-19 in Japan, several implications can be drawn. First, Japan failed to increase the ICU bed capacity in preparation for the COVID-19 pandemic. The number of ICU beds per 100,000 population in Japan was 5.7 in 2020, which is lower than that in other countries, and an increase in ICU beds has been anticipated since the beginning of the COVID-19 pandemic. To strengthen the healthcare delivery system for COVID-19, the Japanese government invested a total of 6 trillion yen to secure hospital beds and medical personnel [28]; however, this did not lead to an increase in critical care recourses. Therefore, the expansion of the ICUs could not have been accomplished simply by investing money; some other methods might have been necessary. Second, in reducing ICU demand in preparation for the COVID-19 pandemic, there was no change in clinicians’ decision-making regarding ICU/HDU admission and discharges, which is in contrast to the recommendations by the guidelines to suspend elective surgery and ICU admissions related to them or discharge ICU patients earlier. This might be attributed to the fact that Japan did not issue guidelines or statements on ICU triage during the COVID-19 pandemic as other countries did [26, 27]. Third, centralization of critically ill COVID-19 patients did not occur in Japan. For a hospital to accept critically ill COVID-19 patients, it required a large fixed budget, regardless of the number of patients. This suggests that it is more efficient to concentrate patients in large hospitals to achieve economies of scale [24, 25]. However, the Japanese healthcare system is characterized by “few large hospitals and many small hospitals”, which makes centralization difficult. This highlights the necessity of designing incentives to promote inter-hospital transport in the future in anticipation of any pandemic or healthcare crisis.

### Strengths and limitations

The present study had several strengths. First, it successfully created a nationally representative cohort of a large number of ICU and HDU patients, representing 70% of all ICU beds and 60% of all HDU beds in Japan. To the best of our knowledge, this is the first study to compare critical care utilization in Japan on a national scale before and during the COVID-19 pandemic. Second, this study is specific to the intensive care field. Critically ill COVID-19 patients need advanced medical treatment such as respiration and circulation. To examine and describe the ICU and HDU bed occupancy and capacity are useful in national critical care system.

The present study had several limitations. First, the calculated occupancy rates depended on the number of ICU and HDU beds that were reimbursed through insurance claims. However, the cost of ICU and HDU stays can be billed only for up to 14 days and 21 days, respectively, and up to 35 days for COVID-19 patients with ECMO. Thus, the calculated occupancy rates in our study may be underestimated. Second, the number of ICU and HDU beds may have fluctuated depending on the burden of COVID-19 and differed from that reported number in the Survey of Medical Institutions 2020, which may have underestimated or overestimated occupancy rates. Third, because the beginning of the COVID-19 pandemic was not clear and differed among prefectures within Japan, the definition of before and after the COVID-19 pandemic using February 9, 2020, as the identified breakpoint in this study may have biased the results of the interrupted time-series analyses. Fourth, although population-based, our study from Japan reflects a single country and may not be generalizable to other countries.

## Conclusion

Using a nationwide inpatient database, we revealed that the ICU and HDU bed capacity did not increase during the COVID-19 pandemic in Japan, whereas their occupancy decreased. There was no change in clinicians’ decision-making to forego ICU care for selected patients, and there was no progress in centralization of critically ill COVID-19 patients. These results will influence healthcare providers, hospital administrators, and policymakers to provide better critical care utilization in anticipation of any pandemics or healthcare crises in the future.

## Supplementary Information

**Additional file 1: Figure S1.** Flow chart of the ICU patients’ selection. **Figure S2.** Daily number of COVID-19 patients treated in ICUs. **Figure S3.** Cumulative number of hospitals treating COVID-19 patients in ICUs. **Figure S4.** Annual hospital case volume of COVID-19 patients in ICUs per year. **Figure S5.** Flow chart of the HDU patients’ selection. **Figure S6.** Daily number of COVID-19 patients treated in HDUs. **Figure S7.** Cumulative number of hospitals treating COVID-19 patients in HDUs. **Figure S8.** Annual hospital case volume of COVID-19 patients in HDUs per year. **Table S1.** Codes used to define ICUs and HDUs using the Japanese medical procedure codes. **Table S2.** Characteristics and outcomes of COVID-19 patients admitted to ICUs and HDUs. **Table S3.** The results of sensitiviry interrupted time-series analysis stratified by the calendar month.

## Data Availability

The dataset analyzed in the current study is not publicly available because of contracts with the hospitals providing data to the database.

## Abbreviations

COVID-19: Coronavirus disease 2019
ICU: Intensive care unit
HDU: High-dependency care unit
DPC: Diagnosis Procedure Combination
ICD: International Classification of Diseases
IMV: Invasive mechanical ventilation
ECMO: Extracorporeal membrane oxygenation
