# Supplementary material for: Impact of the COVID-19 pandemic on critical care utilization in Japan: a nationwide inpatient database study
Source: J Intensive Care. 2022 Dec 2;10:51. doi: 10.1186/s40560-022-00645-0 (PMC9716532; doi:10.1186/s40560-022-00645-0)
Supplement: Supplementary file 1 — Additional file 1: Figure S1. Flow chart of the ICU patients’ selection. Figure S2. Daily number of COVID-19 patients treated in ICUs. Figure S3. Cumulative number of hospitals treating COVID-19 patients in ICUs. Figure S4. Annual hospital case volume of COVID-19 patients in ICUs per year. Figure S5. Flow chart of the HDU patients’ selection. Figure S6. Daily number of COVID-19 patients treated in HDUs. Figure S7. Cumulative number of hospitals treating COVID-19 patients in HDUs. Figure S8. Annual hospital case volume of COVID-19 patients in HDUs per year. Table S1. Codes used to define ICUs and HDUs using the Japanese medical procedure codes. Table S2. Characteristics and outcomes of COVID-19 patients admitted to ICUs and HDUs. Table S3. The results of sensitiviry interrupted time-series analysis stratified by the calendar month. [file 40560_2022_645_MOESM1_ESM.docx]

***Additional File 1***

**Impact of the COVID-19 pandemic on critical care utilization in Japan: a nationwide inpatient database study**

Hiroyuki Ohbe^a^, Yusuke Sasabuchi^b^, Hiroki Matsui^a^, Hideo Yasunaga^a^

^a^Department of Clinical Epidemiology and Health Economics, School of Public Health, The University of Tokyo, 7-3-1 Hongo, Bunkyo-ku, Tokyo, 1130033, Japan.

^b^Data Science Center, Jichi Medical University, 3311-1 Yakushiji, Shimotsuke-shi, Tochigi-ken, 3290498, Japan.

**List of Additional Figures and Tables**

**Additional Figures:**

**Additional Figure S1.** Flow chart of the ICU patients’ selection.

**Additional Figure S2.** Daily number of COVID-19 patients treated in ICUs.

**Additional Figure S3.** Cumulative number of hospitals treating COVID-19 patients in ICUs.

**Additional Figure S4.** Annual hospital case volume of COVID-19 patients in ICUs per year.

**Additional Figure S5.** Flow chart of the HDU patients’ selection.

**Additional Figure S6.** Daily number of COVID-19 patients treated in HDUs.

**Additional Figure S7.** Cumulative number of hospitals treating COVID-19 patients in HDUs.

**Additional Figure S8.** Annual hospital case volume of COVID-19 patients in HDUs per year.

**Additional Tables:**

**Additional Table S1.** Codes used to define ICUs and HDUs using the Japanese medical procedure codes.

**Additional Table S2.** Characteristics and outcomes of COVID-19 patients admitted to ICUs and HDUs.

**Additional Table S3.** The results of sensitiviry terrupted time-series analysis stratified by the calendar month.

**Additional Figure S1.** Flow chart of the ICU patients’ selection.


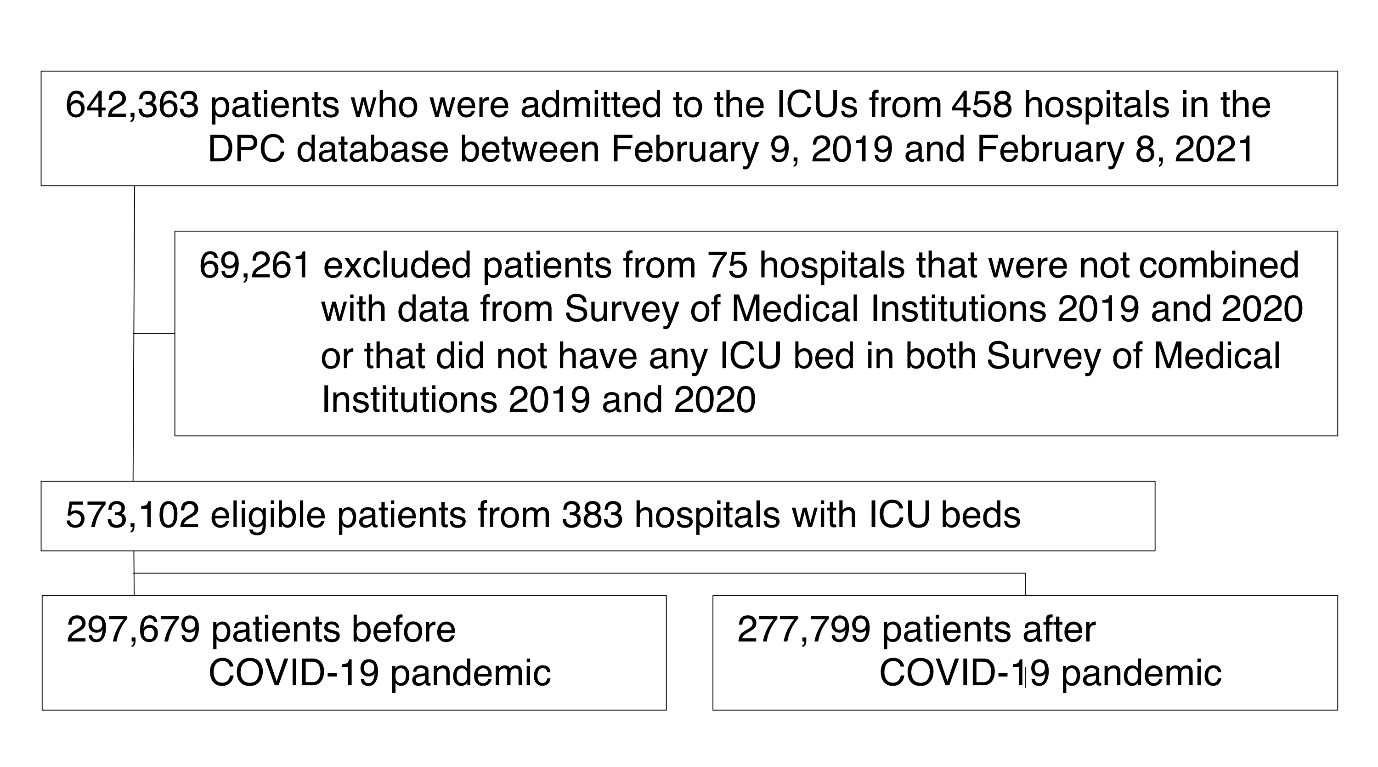


ICU, intensive care unit; DPC, Diagnosis Procedure Combination; COVID-19, coronavirus disease 2019

**Additional Figure S2.** Daily number of COVID-19 patients treated in ICUs.


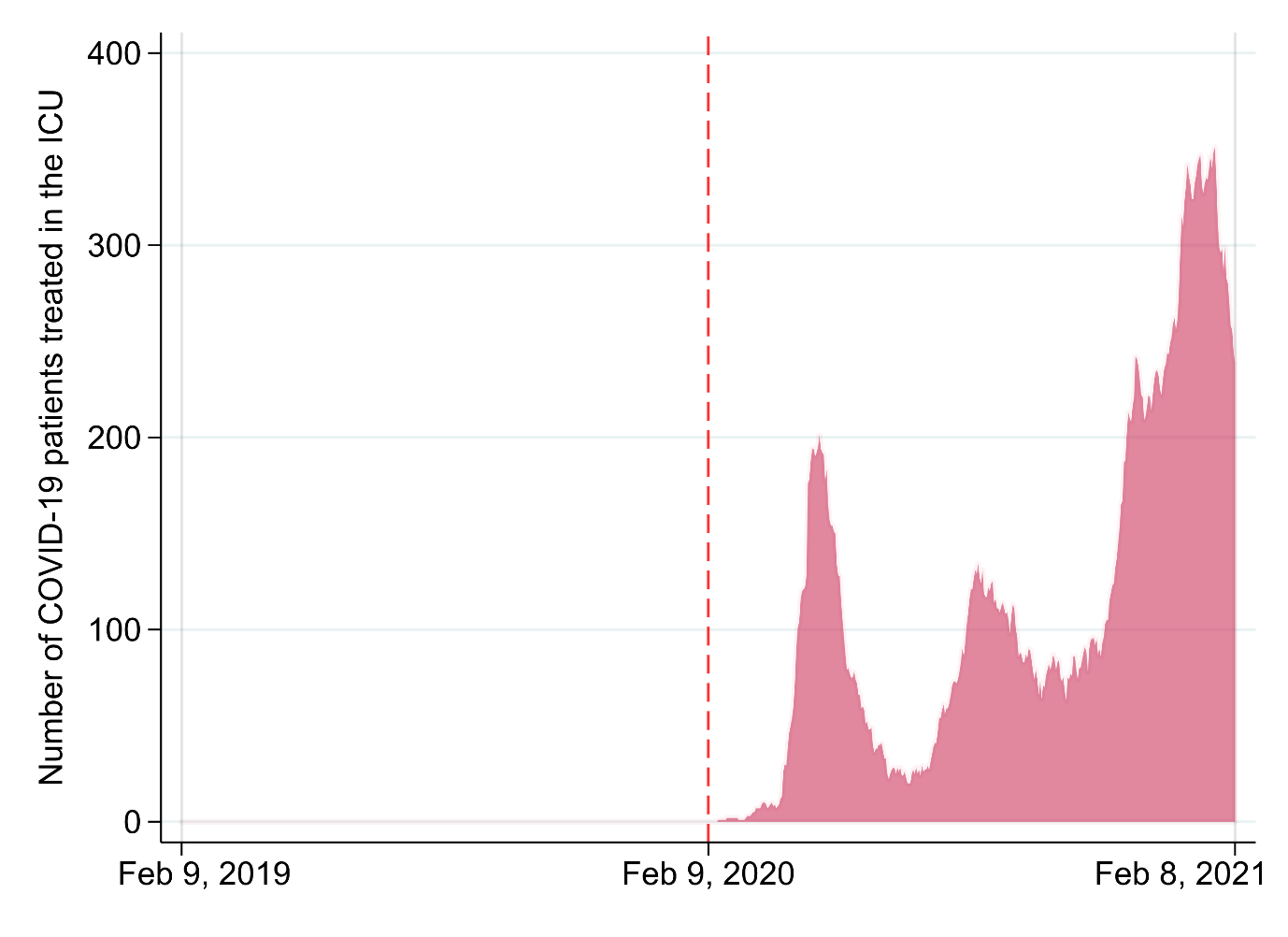


COVID-19, coronavirus disease 2019; ICU, intensive care unit

Red dashed line indicates the start of COVID-19 pandemic on February 9, 2020.

**Additional Figure S3.** Cumulative number of hospitals treating COVID-19 patients in ICUs.


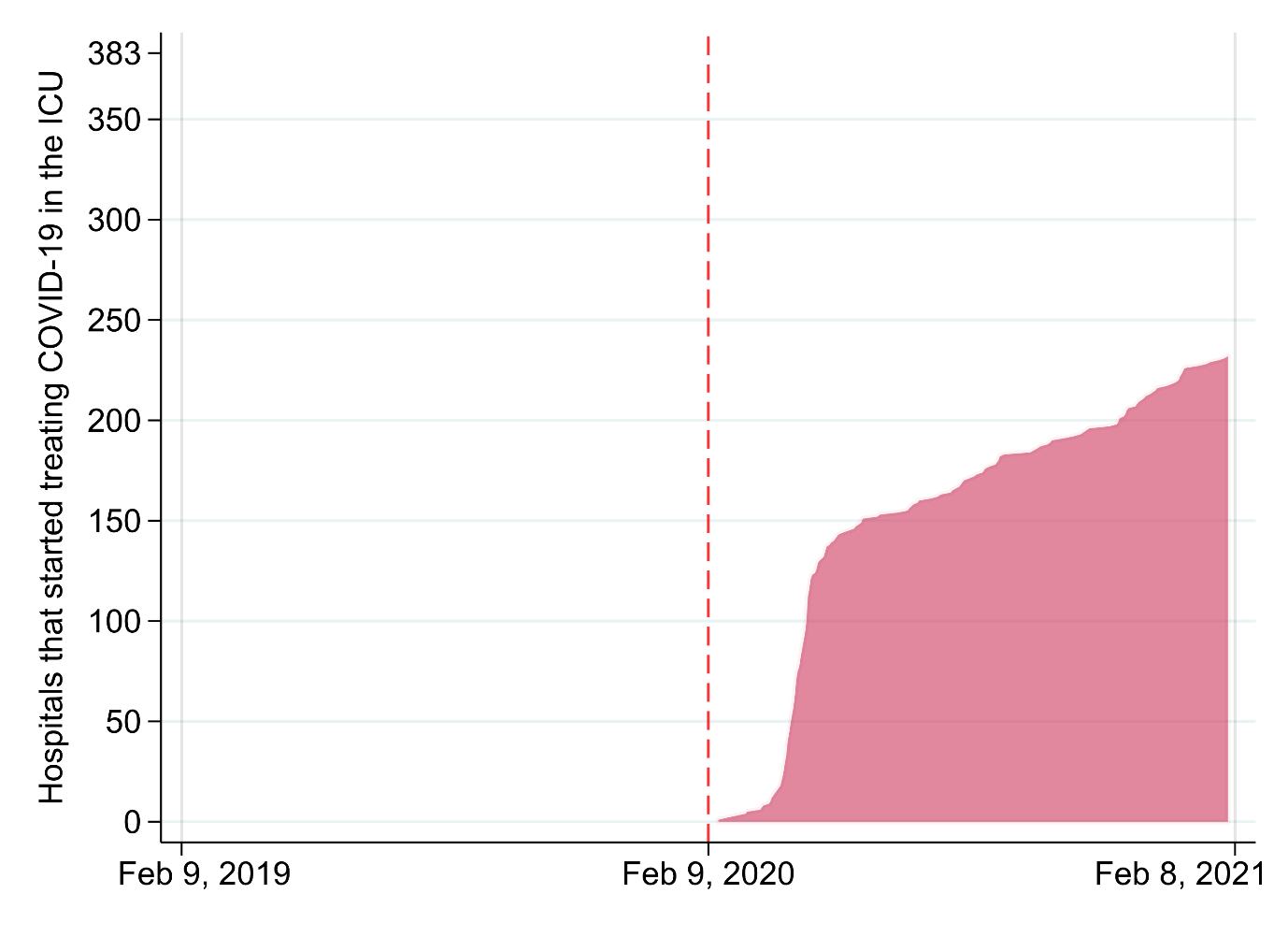


COVID-19, coronavirus disease 2019; ICU, intensive care unit

Red dashed line indicates the start of COVID-19 pandemic on February 9, 2020.

**Additional Figure S4.** Annual hospital case volume of COVID-19 patients in ICUs per year.


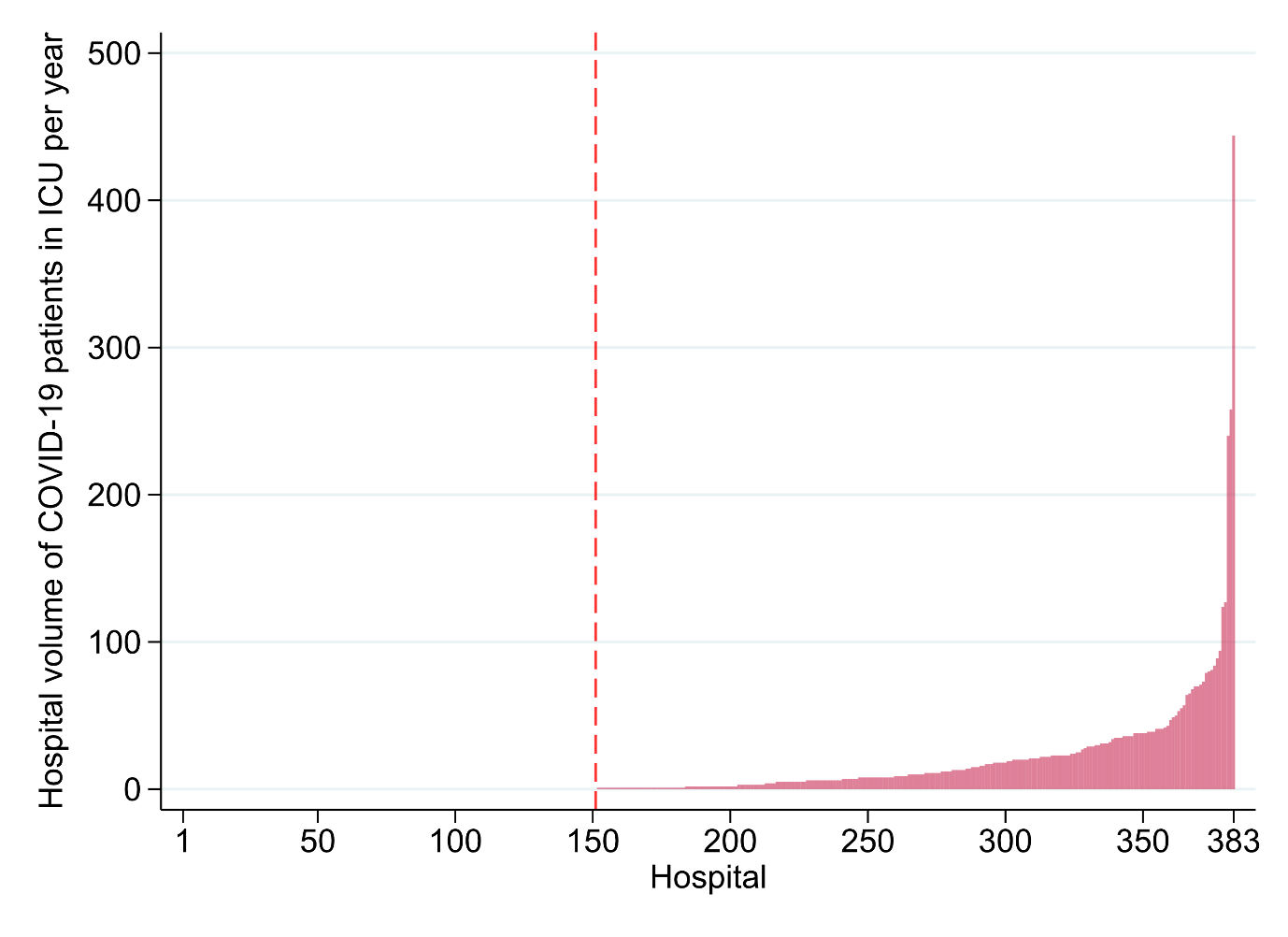


COVID-19, coronavirus disease 2019; ICU, intensive care unit

Red dashed line indicates the breakpoint of whether hospitals treated COVID-19 patients in their ICUs or not within one year since the COVID-19 pandemic.

**Additional Figure S5.** Flow chart of the HDU patients’ selection.


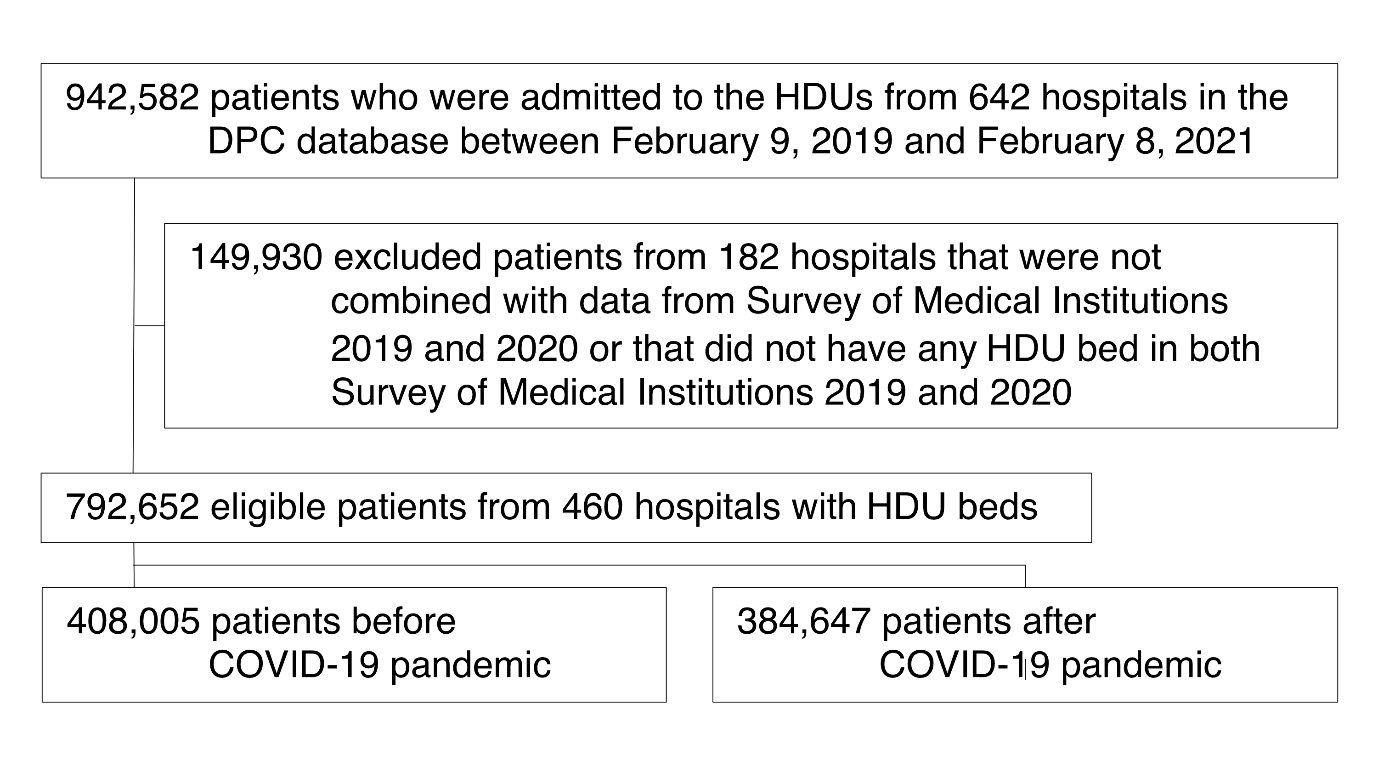


HDU, high-dependency care unit; DPC, Diagnosis Procedure Combination; COVID-19, coronavirus disease 2019

**Additional Figure S6.** Daily number of COVID-19 patients treated in HDUs.


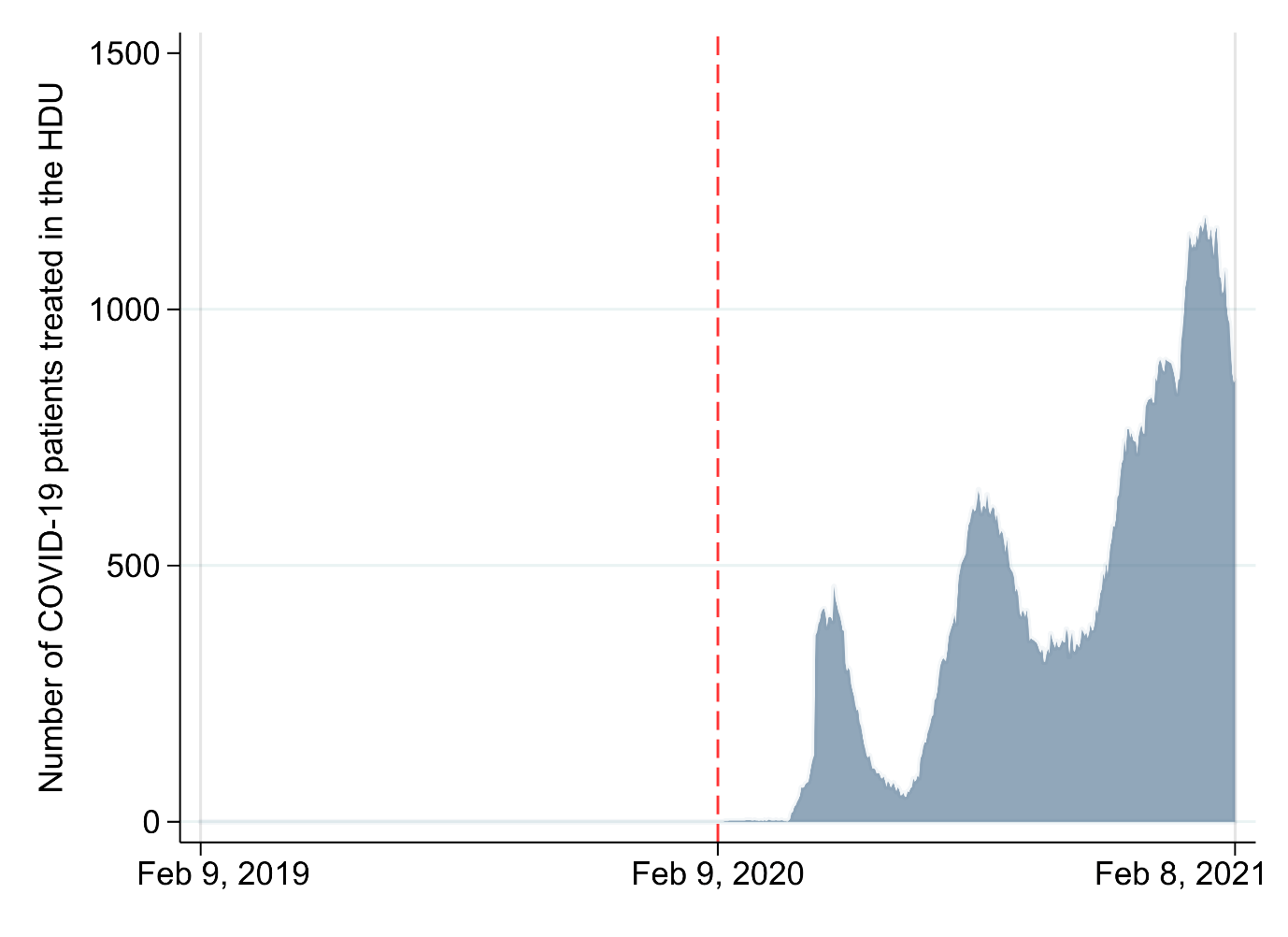


COVID-19, coronavirus disease 2019; HDU, high-dependency care unit

Red dashed line indicates the start of COVID-19 pandemic on February 9, 2020.

**Additional Figure S7.** Cumulative number of hospitals treating COVID-19 patients in HDUs.


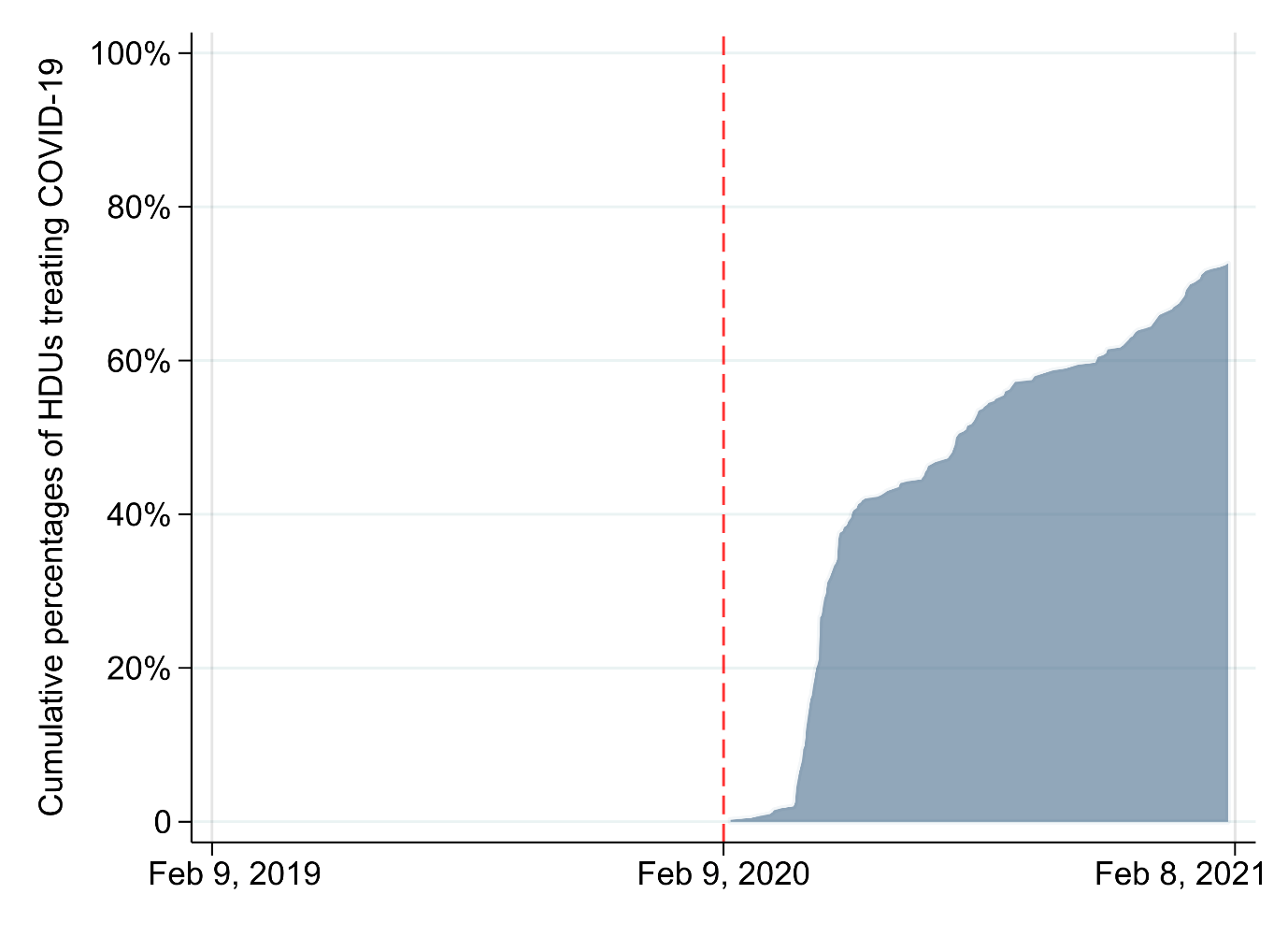


COVID-19, coronavirus disease 2019; HDU, high-dependency care unit

Red dashed line indicates the start of COVID-19 pandemic on February 9, 2020.

**Additional Figure S8.** Annual hospital case volume of COVID-19 patients in HDUs per year.


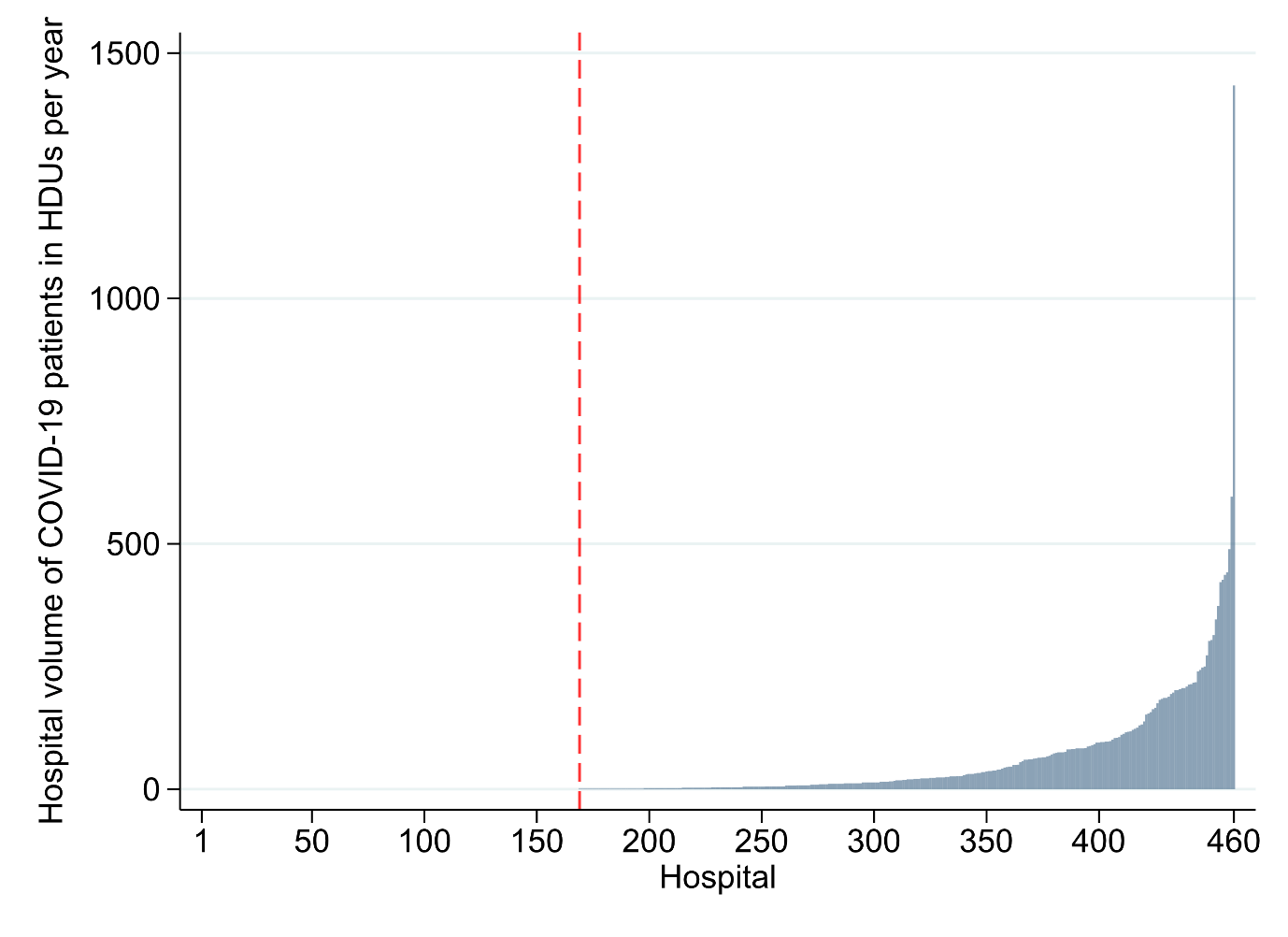


COVID-19, coronavirus disease 2019; HDU, high-dependency care unit

Red dashed line indicates the breakpoint of whether hospitals treated COVID-19 patients in their HDUs or not within one year since the COVID-19 pandemic.

**Additional Table S1.** Codes used to define ICUs and HDUs using the Japanese medical procedure codes.

| Name | Code | Description | Nurse-to-patient ratio |
| --- | --- | --- | --- |
| ICU | A3002 | Emergency and critical care unit management fee 2 | 1:2 |
| ICU | A3004 | Emergency and critical care unit management fee 4 | 1:2 |
| ICU | A3011 | Intensive care unit management fee 1 | 1:2 |
| ICU | A3012 | Intensive care unit management fee 2 | 1:2 |
| ICU | A3013 | Intensive care unit management fee 3 | 1:2 |
| ICU | A3014 | Intensive care unit management fee 4 | 1:2 |
| ICU | A301-4 | Pediatric intensive care unit management fee | 1:2 |
| HDU | A3001 | Emergency and critical care unit management fee 1 | 1:4 |
| HDU | A3003 | Emergency and critical care unit management fee 3 | 1:4 |
| HDU | A301-21 | High care unit management fee 1 | 1:4 |
| HDU | A301-22 | High care unit management fee 2 | 1:5 |
| HDU | A301-3 | Stroke care unit management fee | 1:3 |

ICU, intensive care unit; HDU, high-dependency care unit

**Additional Table S2.** Characteristics and outcomes of COVID-19 patients admitted to ICUs and HDUs.

|  | COVID-19 patients | COVID-19 patients |
| --- | --- | --- |
|  | in ICUs | in HDUs |
| Characteristics and outcomes | (n=5,131) | (n=18,888) |
| Age, year, mean (SD) | 67.1 (16.5) | 61.4 (22.7) |
| Male, % | 3622 (70.6%) | 11188 (59.2%) |
| Route of admission, % |  |  |
| Home | 3409 (66.4%) | 15458 (81.8%) |
| Another hospital | 1566 (30.5%) | 2223 (11.8%) |
| Nursing home | 156 (3.0%) | 1207 (6.4%) |
| Dementia, % |  |  |
| None | 4328 (84.4%) | 15656 (82.9%) |
| Mild dementia | 424 (8.3%) | 1497 (7.9%) |
| Moderate to severe dementia | 379 (7.4%) | 1735 (9.2%) |
| Admission type, n (%) |  |  |
| Elective surgery | 4 (0.1%) | 4 (0.0%) |
| Emergency surgery | 46 (0.9%) | 91 (0.5%) |
| Nonsurgical/acute medical problem | 5081 (99.0%) | 18793 (99.5%) |
| Organ support during ICU/HDU stay, n (%) | |  |
| Invasive mechanical ventilation | 2315 (45.1%) | 1226 (6.5%) |
| Noradrenaline | 1670 (32.5%) | 780 (4.1%) |
| Continuous renal replacement therapy | 378 (7.4%) | 125 (0.7%) |
| Intra-aortic balloon pumping | 17 (0.3%) | 0 (0.0%) |
| Extracorporeal membrane oxygenation | 230 (4.5%) | 40 (0.2%) |
| Left ventricular assist device | 0 (0.0%) | 0 (0.0%) |
| Intracranial pressure monitoring | 3 (0.1%) | 2 (0.0%) |
| ICU/HDU mortality, n (%) |  | 469 (2.5%) |
| Discharge status, n (%) | 332 (6.5%) |  |
| In-hospital mortality | 841 (16.4%) | 1431 (7.6%) |
| Home | 2632 (51.3%) | 13607 (72.0%) |
| Another hospital | 1559 (30.4%) | 2918 (15.4%) |
| Nursing home | 99 (1.9%) | 932 (4.9%) |
| Length of hospital stay, days, median (IQR) | 16 (9-28) | 10 (7-17) |
| Length of ICU/HDU stay, days, median (IQR) | 6 (2-12) | 7 (3-11) |
| Hospitalization costs, million yen, mean (SD) | 2.5 (1.2-5.0) | 1.0 (0.6-1.9) |

COVID-19, coronavirus disease 2019; ICU, intensive care unit; HDU, high-dependency care unit; SD, standard deviation; IQR interquartile range

**Additional Table S3.** The results of sensitiviry terrupted time-series analysis stratified by the calendar month.

|  | Level change, | Trend change, |
| --- | --- | --- |
|  | % | % per month |
| Variables | (95% CIs) | (95% CIs) |
| ICU |  |  |
| ICU bed occupancy, % | -3.80 (-5.32, -2.28) | 0.31 (0.11, 0.51) |
| Invasive mechanical ventilation, % | -2.93 (-3.34, -2.51) | 0.13 (0.07, 0.18) |
| Extracorporeal membrane oxygenation, % | -0.19 (-0.23, -0.14) | 0.00 (0.00, 0.01) |
| Without any organ support, % | -0.68 (-1.56, 0.20) | 0.17 (0.05, 0.29) |
| HDU |  |  |
| HDU bed occupancy, % | -8.24 (-9.21, -7.27) | 1.23 (1.10, 1.36) |
| Invasive mechanical ventilation, % | -1.86 (-2.01, -1.60) | 0.05 (0.02, 0.08) |
| Extracorporeal membrane oxygenation, % | -0.01 (-0.02, 0.00) | 0.00 (0.00, 0.00) |
| Without any organ support, % | -6.21 (-7.03, -5.39) | 1.15 (1.05, 1.26) |

ICU, intensive care unit; HDU, high-dependency care unit; CI, confidence interval
